# Nano‐Encapsulation of Red Beetroot Extract Based on Betanin With Gelatin and Cress Seed Gum by Coacervation Technique

**DOI:** 10.1002/fsn3.71068

**Published:** 2025-10-31

**Authors:** Atefeh Ghoorchi, Akram Arianfar, Vahid Hakimzadeh, Sara Naji‐Tabasi

**Affiliations:** ^1^ Department of Food Science and Technology, Qu.C Islamic Azad University Quchan Iran; ^2^ Department of Food Nanotechnology Research Institute of Food Science and Technology (RIFST) Mashhad Iran

**Keywords:** coacervation, cress seed gum, gelatin, nano‐encapsulation, red beetroot

## Abstract

Co‐encapsulation of more than one core material in a single encapsulation system may increase the bioactivity of individual components. Nano‐encapsulation of red beetroot extract based on betanin with gelatin and cress seed gum by Coacervation technique on particle size characteristics, particle size distribution, zeta potential, X‐ray diffraction analysis, infrared spectroscopy, scanning electron microscopy, and emulsion stability were investigated. All analyses were conducted in a completely randomized design. The results showed that the highest and lowest particle size and particle size distribution were related to samples 0.2% and 0.4% cress seed gum, respectively. Negative zeta potential was observed in all three samples. The reason for negative zeta potential can be attributed to the anionic structure of watercress gum. The amount of zeta potential in sample 0.6% cress seed gum has decreased more. In the XRD test, it showed that the appearance of the peak in the same area indicated the structural similarity of all three samples. The highest peak intensity was in sample 0.6% cress seed gum, and the lowest peak intensity was in sample 0.4% cress seed gum. Electron microscope images of nanocapsules showed that sample 0.6% cress seed gum has a relatively smooth matrix compared to two samples 0.2% and 0.4% cress seed gum, which, apart from the observed holes, indicates the formation of nanocapsules with relatively equal sizes. In total, they have formed a smooth matrix. FTIR analysis showed characteristic functional groups in the nanocapsules. The results showed that an increase in cress seed gum concentration in the nanocapsules led to a greater reduction in particle size, achieving a suitable particle size distribution and a decreased zeta potential in Sample C. The development of stability over time yielded better results. The morphological analysis indicated that most nanocapsules were continuously spherical and regular in shape, demonstrating the success of the formulation used. According to the emulsion stability results after 20 days, it seems that the effect of gum concentration on the emulsion stability is very effective, so the highest and lowest emulsion stability is related to samples 0.6% and 0.2% cress seed gum, respectively. The results of the research showed that the sample containing 0.6% cress seed gum has positive effects on the physicochemical and rheological properties of the nanocapsules.

## Graphical abstract

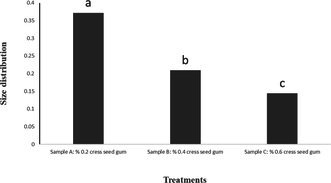

## 1 Introduction

Red beetroot ( *Beta vulgaris* L.) is rich in bioactive compounds like betanin, which gives it a deep red color. Recent studies show it has antioxidant, anti‐inflammatory, and anticancer effects. A 2023 study found that betanin can induce cell death in colorectal cancer cells, highlighting beetroot's potential as a functional food (Kapadia et al. 2023). Red beetroot is a good source of red pigments and is increasingly used for the production of red food colorants. The red and yellow pigments of beetroot are collectively known as betalains and include red betacyanins and yellow betaxanthins. Red beetroot pigments, referred to as betalains, dissolve well in water and are not soluble in alcohol (Neelwarne and Rudrappa 2013). The main pigment in red beetroot is betalain, and it is in the form of glycoside betanidin with a molecular weight of 550.48 g/mol (Neelwarne and Rudrappa 2013). The major and principal betacyanin in red beet is betanin, constituting 75% to 95% of all beet pigments; the remaining pigments include isobetanin, probetanin, isoprobetanin, and others (Azeredo 2009). Due to its rich content of betalains, particularly betanin, and the availability of this plant in our country, its special health benefits, as well as its cost‐effectiveness, beetroot has great potential for use as a source of betalains and a natural food additive in food, pharmaceutical, and health formulations. However, the most significant challenge in using natural food colors is their instability at various stages of production and product preservation (Yousefi 2017).

An emulsion is a system that consists of two immiscible phases in a way that one of the phases is dispersed in the other (Dalgleish 2006). Emulsion systems are typically thermodynamically unstable; therefore, to create a long‐term stable emulsion, emulsifying and stabilizing agents are required (Dickinson 2003). For this reason, in the formulation of emulsion systems, these two components are often present (Dickinson 2008), and most food products contain large molecules such as proteins and polysaccharides that interact with each other and play a significant role in food systems (Benichou et al. 2007). These interactions, which occur in solutions and at common interfaces, are essential for the stability of food dispersions.

The use of nanocapsules for controlled release of bioactive compounds in food materials, such as vitamins, probiotics, bioactive peptides, and antioxidants, has recently gained significant attention (Allen et al. 2006). Various methods exist for encapsulating these nutrients, including spray drying, spray chilling, extrusion, coacervation, co‐crystallization, and freeze‐drying (Li et al. 2009). Coacervation is a relatively simple technique that creates multilayered walls around nanocapsules through electrostatic attraction between oppositely charged components, making them resistant to water and temperature (Li et al. 2009).

Coacervation is a microencapsulation method where a polymer solution separates into two phases, and the polymer‐rich phase forms a coating around core materials, which then solidifies. This technique is commonly used to protect and control the release of active compounds in pharmaceuticals, food, and cosmetics (Khan et al. 2022). The coacervation process has some disadvantages. It is costly and relatively complex. Optimizing the concentration of wall material in emulsification and coacervation processes can be challenging, as the concentration required for forming fine emulsions may differ from that needed to enhance the efficiency of nanocapsules (Azadmard Dimerchi and Emami 2014). Wall materials used are typically polysaccharides and proteins, which are key components in both natural and processed food materials. Such polymers play a significant role in the structure and stability of food systems through gelation, thickening properties, and functional properties in surface stabilization (Lakis 2014). Nanocapsules are mainly formed by polysaccharides and proteins with opposite charges, primarily influenced by the characteristics of broad‐range electrostatic interactions. Physicochemical factors affecting such interactions, such as pH, ionic strength, linear density of polysaccharide charges, surface density of protein charges, hardness and flexibility of the polysaccharide chain, protein size, and the protein‐to‐polysaccharide ratio, have a substantial impact on the formation of these complexes (Nickerson et al. 2018).

One of the critical factors in the production of nanocapsules is the choice of wall material. Polysaccharides and proteins are the two main compounds used in the walls of nanocapsules. Among the polysaccharides used for this purpose are exudate gums. Exudate gums are among the earliest known gums, as they have always been readily available to humans and could easily be collected from trees or bushes. These gums are high molecular weight biopolymers that are used in small quantities in the formulation of food products (Ghasempour et al. 2012). An example of a native gum in Iran is Shahi gum.

The Shahi plant with the scientific name *Lepidium sativum* belongs to the cruciferae family and is commonly known in English as “garden cress”. Garden cress is a small, herbaceous, annual plant that grows to a height of about 50 cm. When its seeds are soaked in water, they quickly absorb water and produce a sticky and tasteless fluid. It has been identified that the seeds contain a significant amount of mucilage compounds. Therefore, hydrocolloid extract from garden cress seeds can provide a suitable market to replace some of the existing hydrocolloids. Given the extensive knowledge of its medicinal uses, such as its anticancer properties, treatment of anemia, and cholesterol‐lowering effects, food products formulated with garden cress seed gum are naturally well received by consumers (Karazhiyan 2010).

Alavi Talab (2012) encapsulated silver carp fish oil using gelatin and Arabic gum. Higher stirring speeds and the use of salt (due to its ionic strength) led to smaller nanocapsules and improved phase separation. Fathi‐Achachlouei et al. (2019) encapsulated wild blackberry anthocyanins using different ratios of gelatin, Arabic gum, and core material. The resulting nanocapsules had lower moisture content, solubility, and hygroscopicity compared to free anthocyanins, with stability increasing by 36% after 2 months. Jafarpour et al. (2017) encapsulated omega‐3 fatty acids using fish gelatin and Arabic gum. Lower homogenization speeds produced smaller nanoparticles.

Despite the increasing interest in natural pigments, no study has yet been conducted on the nanoencapsulation of red beet extract using the complex coacervation method. Considering the health concerns associated with the use of synthetic colorants in food products, this study aimed to enhance the stability of red beet extract through novel nanoencapsulation techniques as a natural alternative to synthetic dyes. The objective of this research was the nanoencapsulation of betalain‐rich red beet extract using the complex coacervation method.

## 2 Materials and Methods

### 2.1 Materials

Red beetroot obtained from the local market. Sunflower oil (Ladan, Iran), PGPR emulsifier (Merck, Germany), Chia seed gum (Rihan Gam Parsian Company, Iran) and Commercial gelatin (Kian Chemistry Company, Iran).

### 2.2 Methods

#### 2.2.1 Determination of the Chemical Composition of Cress Seed Gum

Moisture content, fat, protein, and ash content of cress seed gum was determined using standard AOAC methods with the following numbers: 925.01, 920.85, 920.87, and 923.03, respectively (AOAC 2005). The protein content was determined using an automatic Kjeldahl method (Behr, Germany), and the fat content was obtained through hexane extraction. The carbohydrate content was calculated from the differences in these values.

#### 2.2.2 Red Beetroot Extract Preparation

The red beetroot was washed and peeled, then dried in a dark place at room temperature for 1 week until it reached a constant dry weight. Subsequently, it was ground into a powder using a mill. To extract the beetroot essence, the obtained beetroot powder was mixed with distilled water at a ratio of 1:10 using a magnetic stirrer (Heidolph model, made in Germany) for 30 min. It was then placed in an ultrasonic bath (Parsonic 30s model, made in Iran) with a frequency of 28 kHz for 20 min without applying heat. The obtained betanin extract was filtered using Whatman filter paper and a vacuum pump (Milipore model, made in France), and then centrifuged (PIT320 model, made in Iran) at 7000 rpm for 15 min (Vulic et al. 2012).

#### 2.2.3 Determination of Extracted Betanin Purity Percentage

To ensure the high purity percentage of the extracted betanin from red beetroot and to accurately calculate the amount of betanin, a standard betanin curve was first plotted. For this purpose, 20 mg of betanin was dissolved in 5 mL of water, and different concentrations of it (200, 400, 800, and 1200 μg/mL) were prepared. The absorption of each of them was measured using a visible light spectrophotometer at a wavelength of 538 nm (the maximum absorption of betanin). A standard betanin curve was plotted, and by substituting the absorption values of the specified concentrations of the prepared betanin powders into the betanin standard curve equation, the amount of betanin was calculated and reported as the purity percentage of the prepared betanin powders (Kaimainen et al. 2015).

#### 2.2.4 Mass Encapsulation and Freeze‐Drying

Initially, a W/O emulsion was formed using a 30% (w/v) red beetroot extract solution and sunflower oil along with 3% of the lipophilic emulsifier polyglycerol polyricinoleate 4150. The primary w/o emulsion was transformed into a W/O/W emulsion by adding gelatin solutions as a hydrophilic emulsifier. Both emulsions (simple and double) were obtained in an ultraturax at 12,500 rpm for 4 min. The stability of both emulsions was evaluated through visual observations and optical microscopy. Subsequently, the solution of red beetroot gel was gradually added to the double emulsion at 40°C using a magnetic stirrer. The pH was adjusted to 4 with a 1 M hydrochloric acid solution at 40°C, and the temperature was gradually reduced to 10°C using a magnetic stirrer. The encapsulated material was held at 7°C for 24 h to facilitate phase separation, and then it was freeze‐dried. Three microcapsule formulations were prepared with different concentrations of the red beetroot gelatin encapsulation agent (at concentrations of 0.2, 0.4, and 6.0 mg/mL) along with 5 g of gelatin and 10 g of the core material (the primary emulsion prepared with red beetroot) as a function of the total polymer mass (Fathi‐Achachlouei et al. 2019). The test formulations are presented in Table 1.

**TABLE 1:** Treatments.

Treatments	Gelatin (g)	Cress gum (mg/mL)	Red beetroot (g)
A	5	0.2	10
B	5	0.4	10
C	5	0.6	10

#### 2.2.5 Measurement of Physicochemical Properties of Nanocapsules

##### 2.2.5.1 Determination of Particle Size, Size Distribution, and Zeta Potential

The particle size, size distribution, and zeta potential were determined using a Dynamic Light Scattering (DLS) instrument (Nano ZS ZEN 3600 model, made by Malvern Company, England). For this purpose, 2 mL of the sample (0.1%) was placed in a cuvette and diluted twice with distilled water. The cuvette was then inserted into the instrument, and the parameters mentioned above were determined using visible light with a wavelength of 633 nm at a temperature of 25 degrees Celsius and a pH of 6 (Martin et al. 2017).

##### 2.2.5.2 X‐Ray Diffraction Analysis

The patterns of the cress gum, gelatin, and the nanocapsules produced under optimal conditions were analyzed using an X‐ray diffractometer (XPert Pro model, Holland) operating at 40 kV and 40 mA. The instrument was equipped with Cu‐Kα radiation with a wavelength of 0.1546 nm and a scattering angle range of 5°–75° at a scanning rate of 0.1° at room temperature (Xiaao et al. 2018).

##### 2.2.5.3 Fourier‐Transform Infrared Spectroscopy

The samples were individually subjected to Fourier‐Transform Infrared Spectroscopy (FTIR) (Perkin Elmer model, America) using a spectrometer equipped with OMNIC FT‐IR software. FTIR spectroscopy was performed over the wavelength range of 500 to 4000 cm^−1^ (Fathi‐Achachlouei et al. 2019).

##### 2.2.5.4 Scanning Electron Microscopy

Scanning Electron Microscopy (SEM) (MIR‐3 model, made in TE‐SCAN company, porteghal) was used to investigate the surface properties and microstructure of the nanocapsules. Before analysis, the samples were coated with gold, and a voltage of 15 kV was employed (Kaushik, Dowling, et al. 2015).

##### 2.2.5.5 Emulsion Stability Assessment

The stability of the emulsions was measured based on their visual appearance. After preparation, the samples were placed inside test tubes and sealed, then stored at 5°C. This stability index was evaluated for the samples after 2, 7, and 20 days according to Equation (1) (Huang et al. 2001).

(1) $$\text{Emulsion stability}=\left(\text{height of emulsion layer}/\text{total height}\right)\times 100\%$$

All experiments were conducted in triplicate. The statistical analysis of the results was performed using analysis of variance (ANOVA) in a completely randomized design, and the means were compared using the Duncan method at a 5% significance level. The statistical analysis was carried out using SPSS 22 software, and Excel (2013) was used for drawing graphs.

## 3 Results and Discussion

### 3.1 Chemical Characteristics of Cress Seed Gum

The chemical characteristics of cress seed gum are presented in Table 2. The yield of cress seed gum was 8.14% (based on dry weight). The identification of polysaccharides ensures its purity, which is reflected in its chemical composition, including measurements of total sugar, moisture, and protein (Brummer and Cui 2005). The moisture content of psyllium seed gum was 4.25%. Natural products like gums may contain excess water, which can promote enzyme activation and the growth of microorganisms at an appropriate temperature. They also contain essential nutrients for molds, insects, and mites, which can lead to gum deterioration, so the moisture level in gums is naturally kept around 15% (Malsawmtluangi et al. 2014). The fat content of psyllium seed gum was 3.29%. The fat content of Persian gum, Balangu Shirazi gum, and almond tree gum was reported as 0.03% (Seyfi et al. 2018), 0% (Farhadi 2017), and 2.8% (Fathi et al. 2016), respectively. The content of ash of cress seed gum was 17.12%. The ash content in Malva leaves gum, xanthan gum, Arabic gum, and guar gum is 2.49%, 1.5%, 1.2%, and 11.9%, respectively (Cui 2005). The amount of ash in gum indicates the presence of biological compounds and mineral substances in the plant (Seyfi et al. 2018). The protein content of cress seed gum was 5.28%.

**TABLE 2:** The yield and the chemical components of cress seed gum (based on dry weight).

Treatment	Protein	Ash	Fat	Moisture	Yeild	Total carbohydrate
Cress seed gum	5.0 ± 28.51	17.1 ± 12.04	3.0 ± 29.07	4.0 ± 25.03	8.0 ± 14.12	71.0 ± 14.17

In a study by Cui and Mazza (1996), the protein content for gum arabic, guar gum, and xanthan gum was reported as 1.8%, 8.2%, and 5.4%, respectively (Cui and Mazza 1996). Farahnaki et al. (2011) examined the gum obtained from psyllium seeds and reported its moisture content as 4.84%, protein content as 5.75%, and ash content as 5.75%. Rincón et al. (2014) studied gum from *Prosopis juliflora* and reported protein content as 0.6% and total sugar content as 98.4% (Rincón et al. 2014). The total carbohydrate content in psyllium seed gum was 71.1%. The amount of carbohydrates in the gum indicates its purity (Fathi et al. 2016). Malsawmtluangi et al. (2014) studied the physicochemical properties of gum obtained from Himalayan wild cherry and reported moisture, ash, protein, and total sugar content in this gum as 9.25%, 2.55%, 2.33%, and 73.72%, respectively (Malsawmtluangi et al. 2014). Farhadi (2017) reported the total carbohydrate content in Balangu seed gum as 77.1% (Farhadi 2017).

### 3.2 Determination of the Purity Percentage of Extracted Betanin

By plotting the absorbance of the 4000 μg/mL betanin standard powder on the standard betanin curve, the amount of betanin in the prepared powder was found to be 32.67 ± 1.25 mg/L. Consequently, the percentage purity of the prepared betanin powder was 81.35% ± 2.31%. These results indicate a high degree of purity in the extracted betanin, confirming the suitability of the extraction and drying method for betanin.

### 3.3 Particle Size, Size Distribution, and Zeta Potential Measurement

**FIGURE 1:**
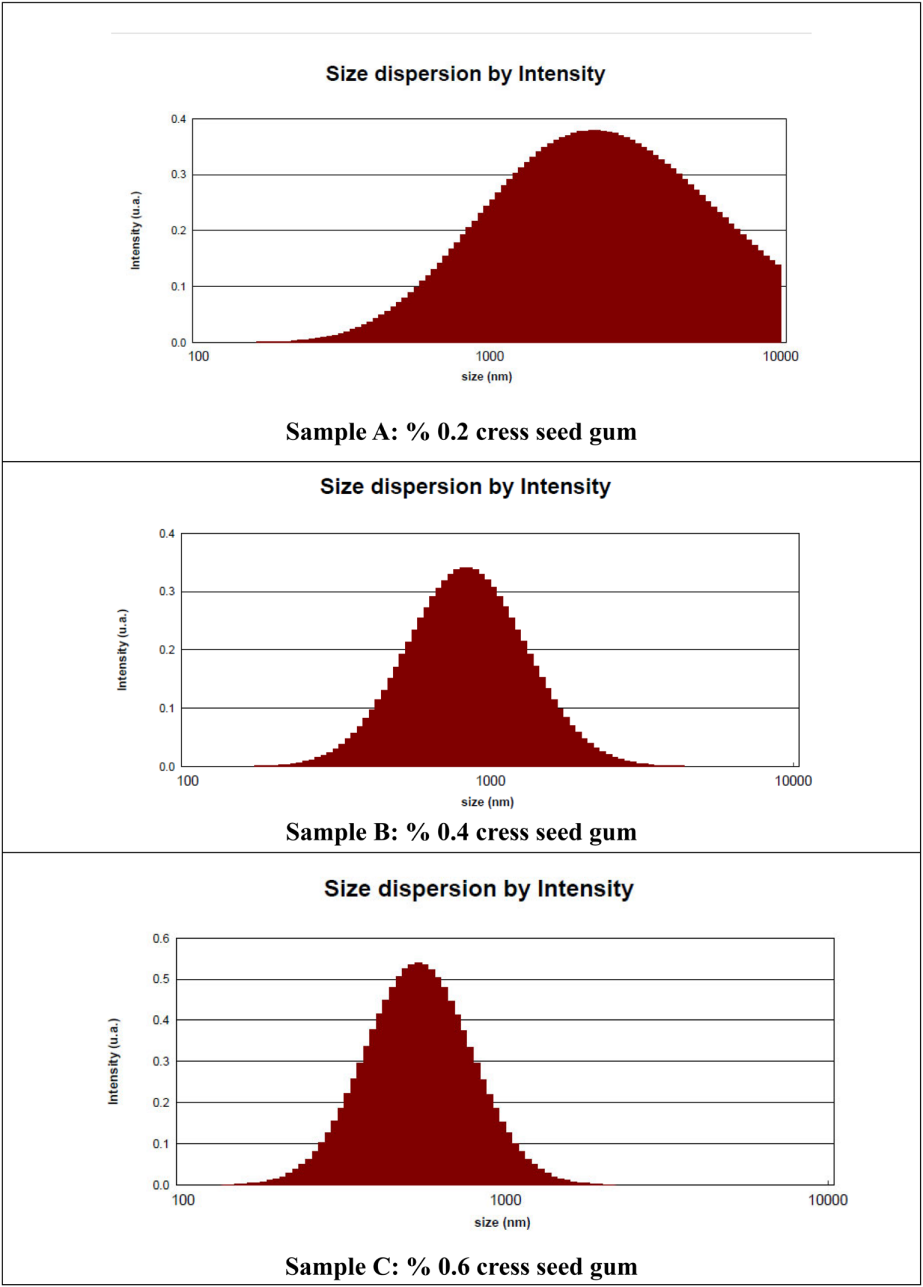
Particle size of nanocapsules measured by DLS.

**FIGURE 2:**
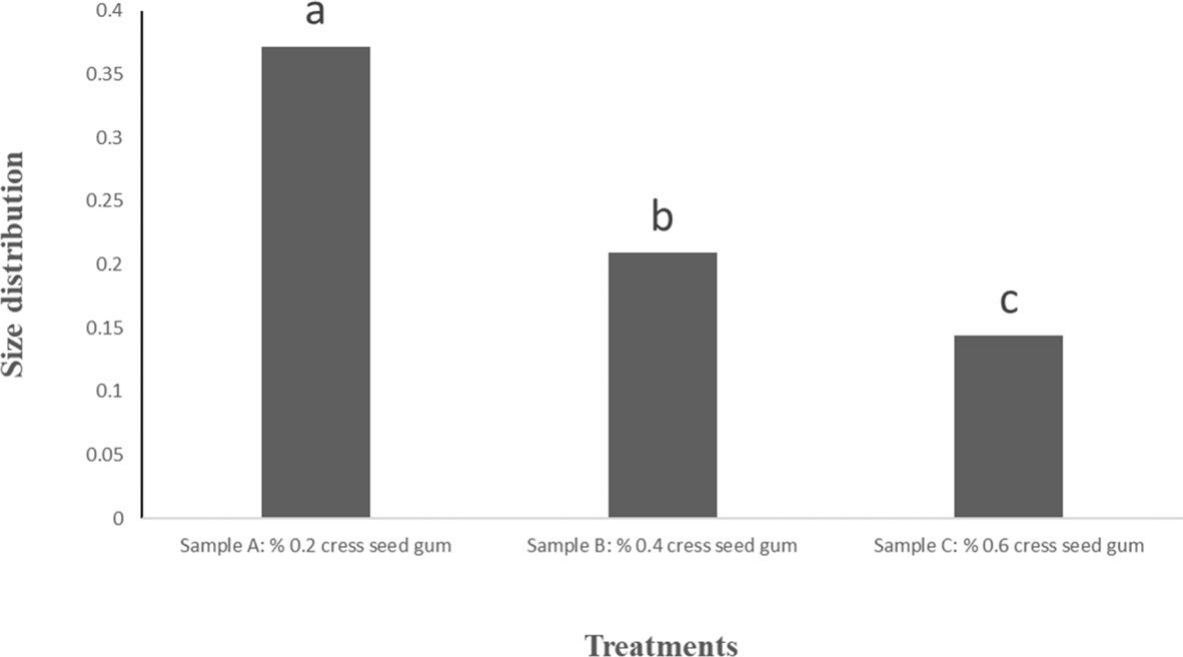
Size distribution of nanocapsules measured by DLS. *Means in a column followed by the different superscripts are significantly different at *p* ≤ 0.05 by Duncan test.

**FIGURE 3:**
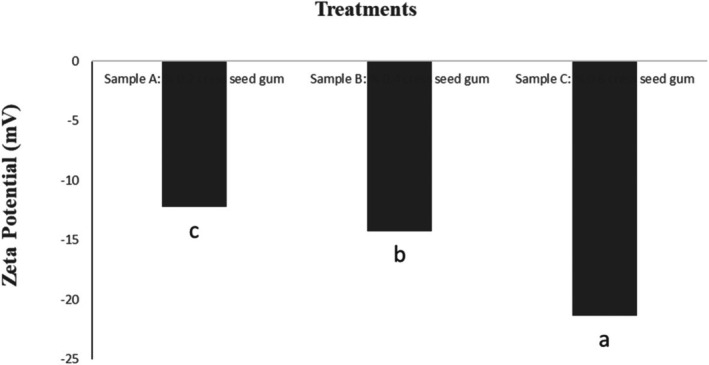
Zeta potential of nanocapsules measured by DLS. *Means in a column followed by the different superscripts are significantly different at *p* ≤ 0.05 by Duncan test.

The particle size, size distribution, and zeta potential are presented in Table 3 and Figures 1, 2, 3. As observed in Table 3, the highest and lowest particle size and size distribution were associated with Sample A and Sample B, respectively. The lower values of these parameters in Sample C indicate that the components are uniformly distributed and, therefore, exhibit better stability. Conversely, higher values of the mentioned parameters indicate non‐uniform distribution and non‐homogeneity of the solution.

**TABLE 3:** Particle size, size distribution, and zeta potential microcapsuls.

Treatments	Partical size (nm)	Size distribution	Zeta potential (mV)
A	1276 ± 0.12^a^	0.372^a^	−12.24 ± 0.25^a^
B	515.01 ± 0.76^b^	0.209^b^	−14.53 ± 0.32^b^
C	330.78 ± 0.25^c^	0.144^c^	−21.37 ± 0.31^c^

*Note:* Means in a column followed by the different superscripts are significantly different at *p* ≤ 0.05 by Duncan test (Sample A: 0.2% cress seed gum, Sample B: 0.4% cress seed gum, and Sample C: 0.6% cress seed gum).

The preparation of a stable emulsion with suitable properties is the most crucial step before the fine coating process (Hosseini et al. 2016). The very small size of droplets provides nanoemulsions with stability against sedimentation and creaming, accompanied by a transparent or slightly cloudy appearance, making them suitable for food applications (Tadros et al. 2004). The particle size and distribution play a crucial role in the physical properties of colloidal systems, such as stability, creaminess, and rheological properties (Azizanbari et al. 2014). In Soottitantawat et al.'s study (2005), it was reported that the particle size of the emulsion has a significant impact on maintaining flavor substances (Soottitantawat et al. 2005).

Zeta potential (ζ‐potential) is a critical parameter for assessing the stability of colloidal dispersions, including nanocapsules. It quantifies the electrostatic repulsion between similarly charged particles in a suspension. High absolute values of zeta potential (typically above ±30 mV) indicate strong repulsive forces, which help prevent particle aggregation and thus confer stability to the system (Bhattacharjee 2016). In this study, the observed decrease in zeta potential in Sample C suggests a reduction in electrostatic repulsion between particles. While this might initially imply a tendency toward instability due to potential aggregation, it's essential to consider other stabilizing factors. For instance, the uniform spherical morphology and reduced particle size observed in Sample C can enhance stability through steric hindrance, where the physical presence of particles prevents them from coming too close and aggregating. Additionally, the presence of specific functional groups from cress seed gum may contribute to steric stabilization (Liu et al. 2015). Therefore, while zeta potential is a valuable parameter for assessing colloidal stability, it should be interpreted in conjunction with other factors such as particle size, distribution, and morphological characteristics to obtain a comprehensive understanding of the system's stability (Bastos et al. 2020).

Beyond electrostatic interactions, steric stabilization plays a vital role in nanoparticle stability. This involves the use of polymers or surfactants that adsorb onto the nanoparticle surface, creating a physical barrier that prevents particles from coming into close contact and aggregating. For instance, coatings like polyvinylpyrrolidone (PVP) have been shown to provide steric stabilization, maintaining nanoparticle dispersion even under varying pH conditions (Kumar et al. 2022).

Influence of Particle Size and Morphology Particle size and morphology significantly influence nanoparticle stability. Smaller particles with uniform, spherical shapes tend to have more stable dispersions due to reduced sedimentation rates and uniform interaction potentials. Studies have demonstrated that nanoparticles with controlled sizes and shapes exhibit enhanced stability, which is crucial for applications in drug delivery and other biomedical fields (Zhou et al. 2021).

Environmental Factors Environmental conditions such as pH, ionic strength, and temperature also impact nanoparticle stability. For example, the stability of nanofluids is optimal at pH values between 4 and 9, and controlling ionic concentration can improve repulsion between nanoparticles, enhancing stability. However, temperatures above 60°C can adversely affect the stability provided by surfactants (Mohammadi et al. 2023). In summary, nanoparticle stability is governed by a combination of electrostatic and steric factors, particle characteristics, and environmental conditions. A comprehensive understanding and control of these parameters is essential for the effective application of nanoparticles in various fields (Ghosh et al. 2022).

Particle size distribution is a molecular parameter commonly used to assess the uniform distribution of molecular sizes (Kang et al. 2011) and is an essential parameter for predicting hydrodynamic volume and rheological properties of biopolymers in aqueous environments. It is also used to ensure the homogeneity and distribution of molecular weight of biopolymers (Bhattacharjee 2016). Particle size distribution less than 0.1 refers to monodisperse solutions, and particle size distribution greater than 0.1 refers to polydisperse solutions (Ma et al. 2017). The size of nanocapsules for food applications, to prevent oral sensitivity to food ingredients, should be less than 100 μm. Additionally, for product integrity preservation, the particle size distribution should be limited (Kaushik, Dowling, et al. 2015).

Akrami et al. (2016) observed that in the production of sodium caseinate‐arabic gum capsules, particle size decreased with an increase in arabic gum concentration up to 0.5%. However, with a further increase in arabic gum concentration, the particle size increased. Wang et al. (2017) also demonstrated that adding carboxymethyl chitosan to zein samples containing beta‐carotene played a cross‐linking role, leading to a reduction in particle size. Luo et al. (2011) showed that in the production of double‐walled capsules containing tocopherol with zein and chitosan, the particle size decreased with an increase in zein content up to 15 mg, but it increased with a further increase in zein content to 20 mg.

Zeta potential is a useful indicator for determining the type and extent of electrostatic interactions in biopolymer systems (Behbahani et al. 2016). The surface charge of the particles is referred to as zeta potential. High zeta potential values result in strong electrostatic repulsion forces and, consequently, enhanced physical stability of the system (Ma et al. 2017). Various factors, including pH, ionic strength, the type and concentration of macromolecules such as polysaccharides, influence the surface charge and zeta potential (Timilsena et al. 2015). As shown in Table 3, all three samples exhibit negative zeta potential values. The negative zeta potential can be attributed to the anionic structure of betanin in the cress seed gum. The zeta potential of cress seed gum is approximately −10.78 mV (Nerkar and Gattani 2012). The zeta potential in Sample C has decreased more. The inhibitory ester is one of the most important factors that contribute to the stability of nanoemulsion particles during storage (Freitas and Müller 1998).

### 3.4 X‐Ray Diffraction

**FIGURE 4:**
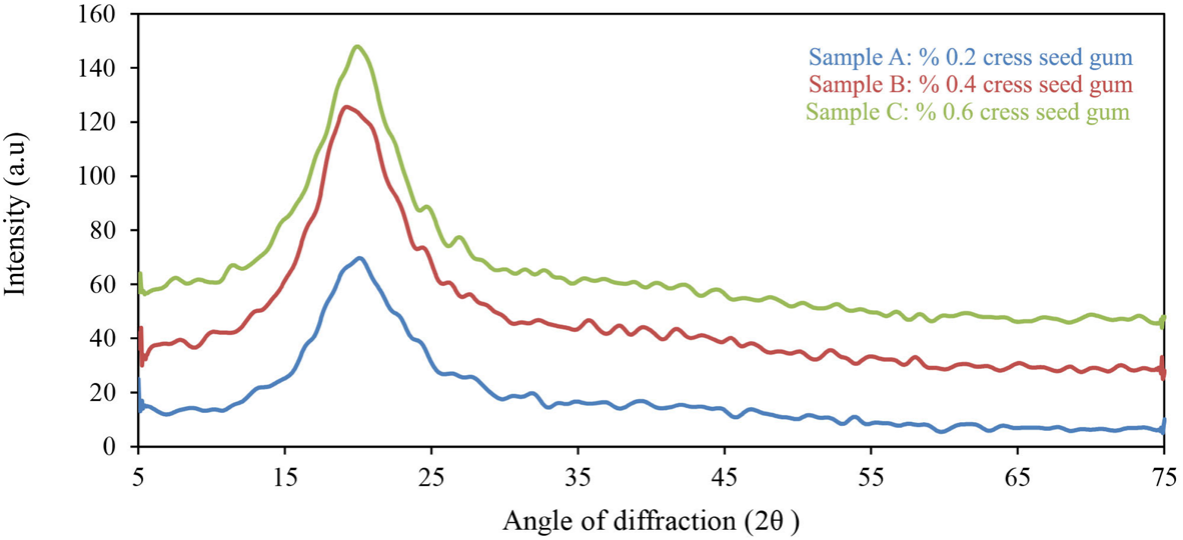
Diffraction X‐ray of nanocapsules.

X‐ray diffraction (XRD) is a technique used to determine the crystalline structure of nanocapsules. Figure 4 shows the XRD spectra of the microcapsule samples. In Sample A, the X‐ray beam angle corresponded to 2θ, and peaks were observed in the 20.14° region. Sample B exhibited peaks in the 19.46° region, and Sample C had peaks in the 20.54° region. The presence of peaks in similar regions indicates structural similarity among all three samples. Sample C had the highest peak intensity, while Sample B had the lowest. In a study by Kaushik, Swami, et al. (2015), on flaxseed gum, a crystalline region was observed at 22°.

One of the methods to determine the crystalline or amorphous nature of a material is X‐ray diffraction. In general, materials with a crystalline structure exhibit sharp and distinct peaks in their XRD graph, whereas materials with an amorphous structure have broader and more diffuse patterns (Caparino et al. 2012).

### 3.5 FTIR Analysis of Nanocapsules

**FIGURE 5:**
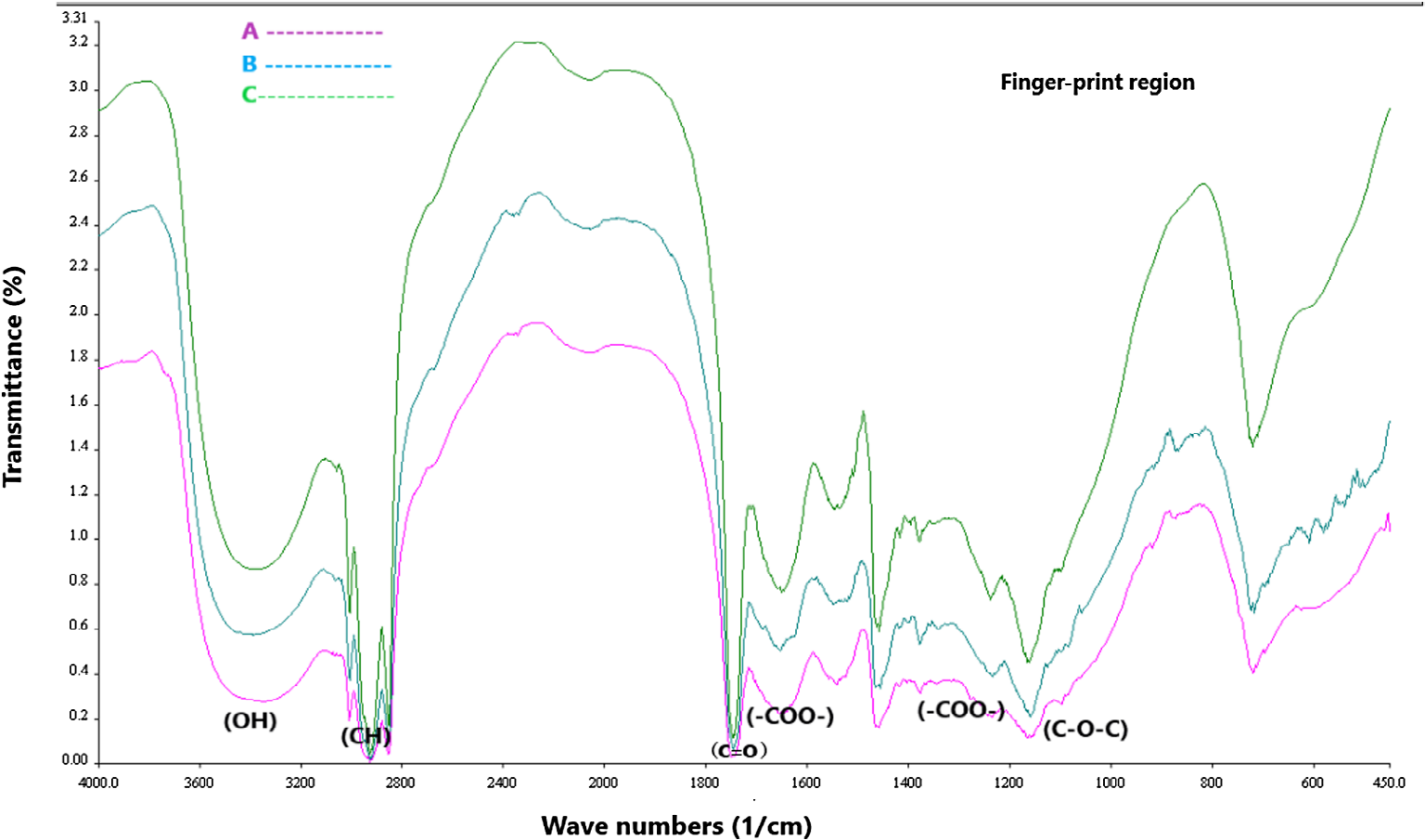
FTIR spectroscopy of the nanocapsules.

Figure 5 shows the FTIR spectra of the nanocapsules. The purpose of conducting the FTIR test is to obtain the chemical spectrum and observe the functional groups in the samples. The peaks in the region of 800–1200 cm^−1^ are referred to as the “fingerprint region” and provide a good indicator of structural differences among different gums (Gan et al. 2004).

No differences were observed between the three samples in the FTIR spectrum (Figure 5). In all three samples A, B, and C, the peaks at approximately 3400 cm^−1^ are attributed to the stretching vibration of hydroxyl (OH) groups attached to carbon, which constitute the main structure of carbohydrates (El‐Aziz et al. 2016). The absorption of OH stretching vibrations leads to a broad absorption region between 3000 and 3500 cm^−1^, which includes various features such as free hydroxyl groups in the vapor phase and O—H bonds in carboxylic acid (Kang et al. 2011).

The wide absorption band with strong intensity between 2800 and 3000 cm^−1^ corresponds to CH stretching vibrations, including CH, CH_2_, and CH_3_ stretching vibrations (Kang et al. 2011). According to Figure 5, all three samples, A, B, and C, exhibit peaks around 2900 cm^−1^, which are attributed to CH stretching vibrations. The range between 1500 and 1800 cm^−1^ indicates the presence of carboxylic acid groups, which belong to the organic acid groups in the gum's structure (Timilsena et al. 2015).

In all three samples A, B, and C the peak at around 1750 cm^−1^ is associated with the stretching vibration of C=O resulting from carboxylic acid ester groups. The carboxylic groups can act as binding sites with ions such as calcium, and these bindings can significantly affect the rheological properties of the gum, such as gel formation and viscosity (Farhadi 2017). Peaks between 1000 and 1200 cm^−1^ result from strong vibrations of —C—O—C— and OH in polysaccharides (Manhivi et al. 2018). The absorption at 1180 cm^−1^ in all three samples, A, B, and C, is attributed to bending vibrations of the pyranose ring and is related to vibrations of C=O, —C—O—C— glucosidic, and C—O—H bonds (Alizadeh Behbahani et al. 2017). Jouki et al. (2014), reported a similar FTIR spectrum for extracted date gum.

### 3.6 Electron Microscopy

**FIGURE 6:**
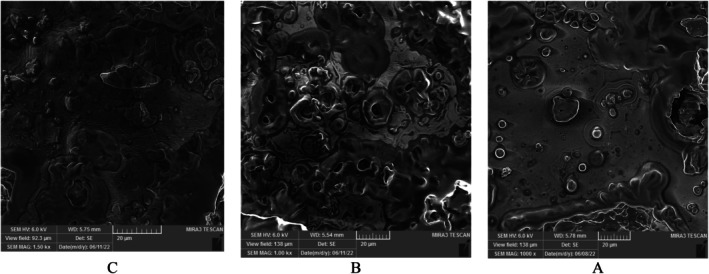
Electron microscopy images of SEM nanocapsules (Sample A: 0.2% cress seed gum, Sample B: 0.4% cress seed gum, and Sample C: 0.6% cress seed gum).

The electron microscopy images of the nanocapsules are presented in Figure 6. As seen in Figure 6, Sample C has a relatively smooth matrix compared to samples A and B. Apart from the observed voids, it indicates the formation of nano‐capsules with relatively similar sizes that collectively make up a smooth matrix. In other words, it suggests the efficiency of the technique used in producing betanin nanocapsules using gelatin and cress seed gum as wall materials at different treatment levels. The obtained images from different treatments show the presence of micro‐pores and fine voids on the matrix's surface, which are a result of ice crystal sublimation.

SEM images can directly provide necessary information regarding the particle size and morphology of the samples (Figure 6). Microscopic observation of the products dried by freeze‐drying serves as a direct way to visualize the matrix structure and, on the other hand, provides evidence of maintaining uniformity and nanoparticles while detecting any changes in their structure (Abdelwahed et al. 2006).

Klinkesorn et al. (2006), in a study on spray‐dried tuna oil, also observed some voids within each capsule, which are likely formed during the final drying phase due to non‐uniform material wrinkling (Klinkesorn et al. 2006). The results of the current study are similar to those obtained in a study by Farias et al. (2007) and Heinzelmann et al. (2000) who observed a matrix with a smooth and porous surface with numerous openings of different sizes in their samples (Farias et al. 2007; Heinzelmann et al. 2000).

### 3.7 Emulsion Stability

The results of emulsion stability after 20 days are presented in Table 4. Based on the emulsion stability results after 20 days, it seems that the concentration of gum has a significant impact. The highest and lowest emulsion stability, respectively, belong to Samples C and A (Table 4).

**TABLE 4:** The results of emulsion stability of microcapsules.

Treatments	Keeping time (day)		
	2	7	20
A	97.28 ± 0.40^b^	53.11 ± 0.38^c^	36.42 ± 0.23^c^
B	100.00 ± 0.00^a^	73.68 ± 0.53^b^	44.69 ± 0.17^b^
C	100.00 ± 0.00^a^	78.56 ± 0.26^a^	45.27 ± 0.18^a^

*Note:* Means in a column followed by the different superscripts are significantly different at *p* ≤ 0.05 by Duncan test (Sample A: 0.2% cress seed gum, Sample B: 0.4% cress seed gum, and Sample C: 0.6% cress seed gum).

The instability of the emulsion at a low concentration of gum can be attributed to the free movement of oil droplets in the system, leading to droplet coalescence. In this concentration of gum, the weak viscous forces in the aqueous phase are not sufficient to prevent droplet collisions, leading to droplet aggregation (Sun and Gunasegaram 2009). Over time, due to increased droplet‐droplet, floccule‐droplet, and floccule‐floccule collisions, the amount of coalesced droplets increases (Chanamai and Mcclements 2002). Furthermore, it appears that at higher gum concentrations, the ability of the gum to cover a significant amount of small droplets is reduced. This is because, at higher concentrations, the gum molecules tend to interact with each other and form larger networks, limiting their ability to cover more droplets. In contrast, at lower concentrations, the gum present, due to the absence of large networks, can cover more droplet surfaces (Beheshti 2010).

The results from Table 4 support this observation, as the droplet size and size distribution in samples A and B are larger than in Sample C, which affects the emulsion separation rate. In this regard, similar results were reported by Khalloufi et al. (2008) and Sun et al. (2007), who investigated the effect of anionic gum from the Tragacanth plant and xanthan gum on the stability of emulsions containing isolated whey protein. They observed similar behavioral changes. Moreover, the phase separation process in emulsion samples occurs at a lower speed with higher gum concentration, and it appears that the high gravity effect of the continuous phase in the presence of gum molecules in the aqueous phase is the cause of this change (Sciarini et al. 2008; Chanamai and Mcclements 2002).

## 4 Conclusion

In this study, the nanoencapsulation properties of red beetroot extract containing betanin, gelatin, and cress seed gum were investigated using the coacervation technique. The results demonstrated that increasing the concentration of cress seed gum led to a significant reduction in particle size, a more desirable particle size distribution, and a decrease in zeta potential in Sample C. Stability also improved over time. Morphological analysis confirmed that most nanocapsules were spherical and uniform in shape, indicating the success of the formulation. The sample containing 0.6% cress seed gum showed enhanced physicochemical and rheological properties. These improvements in physicochemical stability, reduced particle size, and increased emulsion stability offer valuable opportunities for broad applications in the food industry. The developed nanostructure can act as an efficient carrier for natural pigments and bioactive compounds such as betalain, protecting them from environmental degradation caused by light, heat, and oxidation. Therefore, this technology presents a promising natural alternative to synthetic colorants, supporting the development of healthier and higher‐quality food products. Furthermore, the controlled release capabilities of these nanocapsules allow for the gradual delivery of active ingredients, potentially improving the shelf life, flavor, and nutritional profile of food items. Incorporating such systems into functional foods and dietary supplements could enhance the bioavailability of beneficial compounds. Additionally, the biodegradable and natural composition of cress seed gum makes this nanostructure an eco‐friendly option for smart food packaging, contributing to improved food preservation and safety. Overall, the findings support the potential of this nanoencapsulation technology for innovative applications in the food industry, offering both functional and environmental benefits.

## Author Contributions

**Atefeh Ghoorchi:** funding acquisition (equal), methodology (equal), validation (equal), writing – original draft (equal). **Akram Arianfar:** conceptualization (equal), project administration (equal), supervision (equal), validation (equal), writing – review and editing (equal). **Vahid Hakimzadeh:** project administration (equal), validation (equal), writing – review and editing (equal). **Sara Naji‐Tabasi:** conceptualization (equal), project administration (equal), validation (equal), writing – review and editing (equal).

## Conflicts of Interest

The authors declare no conflicts of interest.

## Data Availability

The data that support the findings of this study are available from the corresponding author upon reasonable request.
